# Integrating Telehealth Care-Generated Data With the Family Practice Electronic Medical Record: Qualitative Exploration of the Views of Primary Care Staff

**DOI:** 10.2196/ijmr.2820

**Published:** 2013-11-26

**Authors:** Emma Davidson, Colin R Simpson, George Demiris, Aziz Sheikh, Brian McKinstry

**Affiliations:** ^1^eHealth GroupCentre for Population Health SciencesThe University of EdinburghEdinburghUnited Kingdom; ^2^Biomedical and Health InformaticsSchool of Medicine & Biobehavioral Nursing and Health Systems, School of NursingUniversity of WashingtonSeattle, WAUnited States; ^3^Division of General Internal Medicine and Primary CareBrigham and Women’s Hospital/Harvard Medical SchoolBoston, MAUnited States; ^4^Edinburgh Health Services Research UnitNHS LothianEdinburghUnited Kingdom

**Keywords:** telehealth care, family practice, data management

## Abstract

**Background:**

Telehealth care is increasingly being employed in the management of long-term illness. Current systems are largely managed via “stand-alone” websites, which require additional log-ons for clinicians to view their patients’ symptom records and physiological measurements leading to frustrating delays and sometimes failure to engage with the record. However, there are challenges to the full integration of patient-acquired data into family physicians’ electronic medical records (EMR) in terms of reliability, how such data can best be summarized and presented to avoid overload to the clinicians, and how clarity of responsibility is managed when multiple agencies are involved.

**Objective:**

We aimed to explore the views of primary care clinicians on the acceptability, clinical utility, and, in particular, the benefits and risks of integrating patient-generated telehealth care data into the family practice EMR and to explore how these data should be summarized and presented in order to facilitate use in routine care.

**Methods:**

In our qualitative study, we carried out semi-structured interviews with clinicians with experience of and naïve to telehealth care following demonstration of pilot software, which illustrated various methods by which data could be incorporated into the EMR.

**Results:**

We interviewed 20 clinicians and found 2 overarching themes of “workload” and “safety”. Although clinicians were largely positive about integrating telehealth care data into the EMR, they were concerned about the potential increased workload and safety issues, particularly in respect to error due to data overload. They suggested these issues could be mitigated by good system design that summarized and presented data such that they facilitated seamless integration with clinicians’ current routine processes for managing data flows, and ensured clear lines of communication and responsibility between multiple professionals involved in patients’ care.

**Conclusions:**

Family physicians and their teams are likely to be receptive to and see the benefits of integrating telehealth-generated data into the EMR. Our study identified some of the key challenges that must be overcome to facilitate integration of telehealth care data. This work particularly underlines the importance of actively engaging with clinicians to ensure that systems are designed that align well with existing practice data-flow management systems and facilitate safe multiprofessional patient care.

## Introduction

### Background

The changing global demography poses the twin challenges of an aging population, who suffer from a high and increasing prevalence of long-term conditions, and the falling numbers of people who can provide care for them. This realization has catalyzed international interest in self-monitoring and self-management of long-term conditions as one possible solution to this problem. Telehealth care, which uses information technologies (IT), to support such self-monitoring has the potential to be particularly useful in this context [1]. It has been used in a wide variety of conditions, principally the management of congestive heart failure (CHF) [2], diabetes mellitus [3], hypertension [4], chronic obstructive pulmonary disorder (COPD) [5], and asthma [6]. In most models of care, patients record symptoms (eg, breathlessness and cough) and physiologic data (eg, weight, blood pressure—BP, peak expiratory flow, and blood glucose). These data are then relayed via the Internet to a central server from where these are made available in a variety of summarized forms to both the patients and clinicians by providing alerts when preset symptom scores or physiologic parameters are breached. Clinicians may view data as needed, for example, daily for less stable conditions such as COPD and CHF or less frequently where there is less likelihood of rapid deterioration such as hypertension or diabetes.

### Telehealth Care Data and Electronic Medical Records

The adoption of new IT systems of care is sometimes met with resistance from health practitioners, this often, at least in part, stemming from fears of increased workload [7,8]. Usability of such systems is paramount in determining if they will be successfully integrated within normal working patterns [9,10]. Throughout the course of our program of randomized controlled trials (RCTs) in telehealth care [11], a recurring issue has been the inability to integrate telehealth care-generated data into the electronic medical records (EMR) of family physicians [12-14]. Data are, therefore, usually stored on a separate website, which necessitates additional security log-ons and, in some cases, double entry of data resulting in lengthening of the consultation [15] and possibly introducing new data security risks. Finding a solution to these issues will become increasingly important as telehealth care systems become more widely deployed. The challenges of interoperability and data integration particularly affect care coordination, which is increasingly viewed as an essential component of patient-centered comprehensive care (reflected in the United States, for example in the concept of the patient-centered medical home) [16,17].

The National Institutes of Health in the United States held a conference in 2009 on the future of telehealth and identified the integration of telehealth data into EMR as a high impact topic that could potentially determine the success and future of telehealth [18]. Systems are now being developed to support integration of telehealth care-generated data into the EMR. However, it is not clear what preference physicians and general practice staff may have in terms of the types of data they would like uploaded into their systems, how these data should be summarized, what data reliability considerations should be considered (eg, the accidental inclusion of erroneous readings, such as improbably low weights, normally ignored by clinicians), and what medicolegal concerns clinicians may have.

We aimed to investigate the views of family physicians and their teams on the acceptability and clinical utility of integrating telehealth care data into EMR. In particular, we sought to understand what they viewed as the risks and benefits of importing such data and how they should be presented and summarized in order to maximize acceptability and thereby facilitate use.

## Methods

### Design

We undertook a qualitative study—through general practices in Edinburgh, Scotland, United Kingdom—which consisted of semi-structured interviews with primary care practice staff following demonstration of pilot software, which illustrated a variety of methods by which data could be incorporated into the EMR.

### Sampling and Recruitment of Practices and Participants

From practices that had been involved in our RCTs of telehealth care monitoring in hypertension and diabetes, a family physician and a practice nurse who were personally involved in telehealth care management were selected. However, we also considered it important to determine the views of family physicians and nurses who may be less familiar with telehealth care technology as those practices who had agreed to take part in telehealth care studies may preferentially have interested “early adopters” [19,20] and any large scale roll-out of telehealth care will need to involve those who tend to embrace such technologies less readily.

We therefore aimed to purposefully sample physicians and practice nurses representing a range of ages from telehealth care experienced and naïve practices, from areas of differing socioeconomic levels, and family practice size. Initial contacts were made through a personal approach to potentially suitable clinicians who had taken part in our telehealth RCTs. We also approached practices who had previously been invited to take part in the RCTs, but had decided not to and also other nonparticipating practices in the Lothian research network. In addition, we interviewed two specialist community respiratory physical therapists who had participated in previous telehealth care research and could provide complementary insights into how the integration of telehealth data into the primary care EMR could impact on the wider multidisciplinary care team.

### Data Generation

The interviewees were shown pilot software developed by the Department of Health’s National Health Service Connecting for Health (CfH) Informatics Directorate Assistive Technology Programme team in association with Newham Primary Care Trust London, which was designed to link patient accrued data from the Philips Motiva [21] telehealth system with the EMIS [22] Web GP EMR system (which is one of the most commonly used systems in the United Kingdom). This was shown as a PowerPoint presentation and animation on a laptop computer. This pilot software enables interoperability between health care systems, allowing telehealth care-generated data (eg, BP readings) to be viewed using the family practice EMR system and then permanently filed into the patient EMR. A full description and screenshots from the system are included in Multimedia Appendix 1. Clinicians who had not been involved with telehealth care were, in addition to the presentation, given details of how a telehealth care system works and given an indication of the quantity and quality of data that are expected to be generated by such systems.

In depth, face-to-face interviews with family physicians and practice staff were carried out at the practices following the software demonstration. Interviews were digitally audio-recorded and transcribed. An initial topic guide (see Multimedia Appendix 2), based on established research on diffusion of innovation in health service organizations [23], was used to aid discussion, and this guide was reviewed and iteratively refined during the process of data collection and analysis.

### Data Analysis

Thematic analysis [24] was used to identify the factors that might influence the integration of telehealth care-generated data into the family practice EMR. Analysis was supported by NVivo. Transcripts were repeatedly read and coded to include both anticipated and unanticipated themes. Analysis was ongoing to allow emerging themes to be fed back into the data collection. Constant comparison was employed to ensure that the thematic analysis represented all perspectives and negative cases were actively sought [25].

A coding framework was drawn up by the research team as new themes emerged. The coding framework was informed by the aims of the research and research questions and previous research in examining the processes whereby telehealth innovations are developed, implemented, and sustained [7,23]. As analysis was ongoing, the content of the latter interviews could be examined against the coding framework (Textbox 1). We limited interviews to 20 as it was clear that no new insights were being generated beyond the 15th interview and saturation had been achieved. The ongoing discussion of the findings among the project team for analysis enhanced the trustworthiness of the findings.

**Figure 1:**
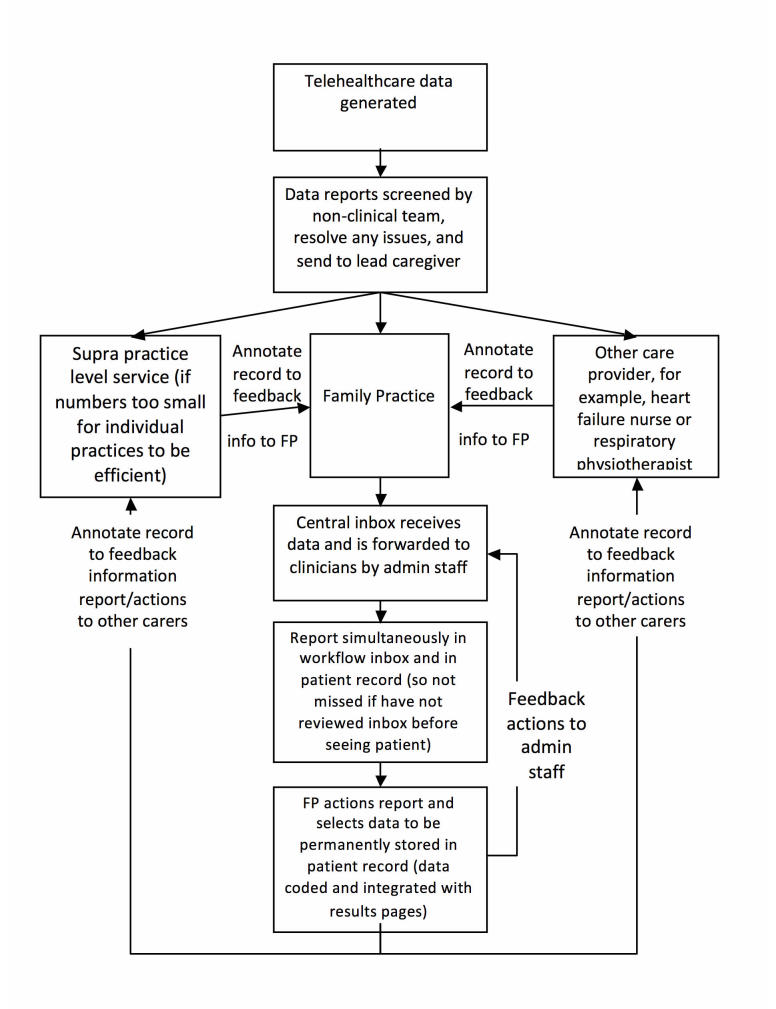
Model data pathway for telehealth integration system.

During the emergence of the data, it became apparent that several of the themes provided an outline of the “optimal” telehealth care system design desired by family physicians and their teams. These data were, therefore, further discussed by the project team and combined to create a model data pathway for a telehealth integration system (Figure 1).

**Thematic organization.**

Perceptions of workload:

System design

Previous experience of telehealth systems

Efficiencies through improved access

Ease of use

Training and support

Data management

Amount of data

Flexibility of data parameters

Data flows

Data coding

Payment

Safety:

Impact on professional-patient relationship

Data quality

Risks of data overload and error

Confidentiality

Liability

## Results

### Participants’ Characteristics and Software Demonstration

The completed dataset comprised 20 participants. We recruited 10/20 (50%) professionals with experience of telehealth care and 10/20 (50%) without any prior experience, 10/20 (50%) were family physicians, 8/20 (40%) practice nurses, and 2/20 (10%) specialist respiratory physical therapists. The participant’s characteristics, including who had experience of telehealth care, are outlined in Multimedia Appendix 3. The software was demonstrated either in the participants’ own home or in workplace according to their preference and the interview took just under an hour.

### Main Themes

#### Overarching Themes

The main themes identified are discussed below and summarized in Textbox 1. In addition to these key themes, there was discussion of the utility of telehealth more generally; however, this is not discussed here as similar findings have been published elsewhere [12,13,26].

There were two overarching themes, which encompassed the barriers and facilitators to integrating patient accrued data. These were:

- *Perceptions of workload* which incorporated the importance of good system design and training, the likely quantity of data that would have to be processed, and if physicians should be given additional payment for overseeing the integrated data.
- *Safety* which encompassed concerns about the possible impact on physician-patient consultation, confidentiality issues, data quality, error due to data overload, interprofessional communication and responsibilities, and, related to these issues, concerns about liability.

#### Perceptions of Workload

##### System Design

###### Previous Experience of Telehealth System Design

Participants with preexisting knowledge of telehealth were generally most positively disposed toward IT and were more enthusiastic about integrating telehealth care data into the EMR. This positive attitude arose because their previous involvement in telehealth was colored by the frustrating and time-consuming experience of having to access an additional website for results. However, other participants described their own lack of IT experience or expertise may deter them from adopting and, in turn, adapting to new systems. For some clinicians, suggestion that they should engage with additional IT posed a seemingly insurmountable barrier.

Some people would absolutely freak out, even some of our colleagues. I don't know if that's the sort of... anybody's said that, because some people are just absolutely at breaking point with regard to using technology and some people it's just the last kind of thing that they can take, do you know what I mean?

Family physician—FP3 Telehealth care naïve—TN

###### Efficiencies Through Improved Access

The pervasive benefit from adopting an integrated system was seen to be improved access to data. When access involved an additional log-on many doctors and nurses (except those directly involved in a trial) did not access these data; however, they felt that with data integrated into the EMR they would definitely utilize it for patient management.

It’s just the fact that you had to then print off all the details from your telehealth and then transfer all those details into the patient’s notes. … So, if it was transferring into the patients’ clinical notes automatically I think it would be great.

Practice nurse—PN9 Telehealth care experienced—TE

Despite concerns over increasing workload, there was recognition of the potential for an integrated telehealth system to reduce consultations and home visits while enhancing patient care.

Certainly going to give you a better picture of really where a patient is sitting at, if you're getting all these current results coming in… What I'm trying to say, you might prevent a whole load of unnecessary visits, medications, if you’re actually getting a clearer picture of where they are.

PN19 TN

###### Ease of Use

Among all participants, it was a priority that any future system is simple to use and terms such as “user friendly” and “intuitive” arose frequently in their discourse. “Reducing the number of clicks... that you need in order to deal with something...” [FP10 TE] was deemed essential.

Yeah, keeping it, probably, keeping it simple, keeping it easily accessible, so, you know, a click away is always what they say for a lot of these things, and clear and concise with not, you know, just keeping the information basic without having to read through screeds and screeds [a lot] of stuff that’s potentially not necessary for what your job entails.

PN15 TE

###### Training and Support

Although the demonstrated system appeared relatively easy to use, training was thought important and this would, as suggested, have to be tailored to individuals’ IT knowledge. Any additional new software for managing the integrated data, including setting preferences for displaying data and warnings, needed to closely resemble the existing EMR as much as possible. It was suggested that if the screen presentation, commands, short-cuts, etc for displaying the data were similar to the EMR, this would reduce the training required. Training could also be assisted by a few members of staff particularly trained as “on-site experts”, who could provide a first line of support to the practice, and by follow-up training sessions scheduled once physicians and nurses have trailed the system.

No, I think you’d try and design it so there was virtually no training required. You’d try and design so that the dataflow was no different to dataflow in the practice... depending on other sources, cause you can make it different...

FP7 TE

##### Data Management

###### Developing a System

Overall, it was felt that barriers to adoption would be greatly reduced by developing a system that was as compatible as possible with existing working practices:

You need to pick a system that does what you want it do, whereas what we were given here [previous telehealth system] was a system and we had to try fit with it. Whereas, actually, the idea of telehealth is it needs to fit with what you’re doing.

PT4 TE

###### Amount of Data

The participants felt it was important that a system should allow choice of exactly what, and how much, data get imported into the EMR as otherwise “you don’t see the wood from the trees” [FP7 TE]. It was considered essential that the EMR was not “cluttered” with telehealth information to the detriment of other clinical information. The amount of data desired also varied according to the disease being monitored and who was the main case manager. They suggested that a filtering facility may be beneficial on the working screen, allowing display of imported data among routine health care consultations, or remove it to view consultations alone. Data summaries were preferred as opposed to raw figures. Therefore, for some, graphical presentation summarizing patient data with easy access to a fuller report from within the EMR were viewed as extremely useful. However, views on the utility of graphical presentation were mixed and related to personal preference. The ability to choose format was therefore desirable. Participants who preferred graphical data described this as a useful aid in consultation with patients.

What primary care wants is the report... but you probably don’t need all the 40 values... and, I suppose, telehealth is no different… you know, what you want is the range... for that person, and somebody can give you the variability.

FP7 TE

###### Flexibility of Data Parameters and Alerts

All clinicians valued the flexibility to set data parameters specific to each patient, which trigger an alert if readings fall outside these limits, with the caveat that parameters were easily set and visible alongside any results they received. There were concerns, however, about who set parameters and lines of responsibility; how often these would need to be updated (eg, when people’s clinical condition or treatment changed); the workload involved; and how all care teams would be alerted when and why they had been altered.

Being able to use the parameters and decide who it's going to, I like that, that you can set it for that. Because every patient is so different and you do worry that there’s just a blanket approach, which there just can't be with patients because they’re so individual—yeah.

FP3 TN

In this context, alerts prompting people to revise data parameters were discussed, with mixed views on their utility. Some clinicians felt that these were unnecessary as they should review settings as part of their usual management. Other clinicians were concerned that such reminders would quickly become an annoyance with the risk that they became immune to them or missed them if they popped up and faded. There was agreement that there should be the option to turn them off.

I think, it could be useful, I think, if you’re going to design that you probably want to design an option to turn the pop-ups on or off… I, personally, don’t like those [referring to fading reminders] because if you’ve turned it on, and you’re talking to the patient, because, and not looking at the screen, you might not see it.

FP17 TN

###### Data Flows

Data flows arose as an important aspect of the system design. Data flow depended on the disease and number of patients being monitored. For most conditions, physicians felt that data should initially be screened by a nonclinical worker (eg, a telehealth care service), to screen out any technical problems (eg, people not taking measurements, faulty equipment, etc) as the workload was too great and not an efficient use of their time.

If you have... a thousand patients on telehealth, it’s not an effective use of clinician time, so, we’re back to the nonclinician following an algorithm of whether to phone, or not… one of the lessons we learnt with telehealth in Lothian was that doing it at a practice level is not very efficient....

GP7 TE

Doctors stressed that those messages requiring “action” should be sent to the lead care provider and those for “information only” sent to the entire care team and clearly labeled as such. A priority was that data, particularly “action” messages, did not go astray.

Yes, there has to be some way of making sure that somebody sees important—yes, I think there has to be some grading of how urgent things are… if you could set parameters above which something would flash up or—but, yes, the reception staff who are directing things would sort that out.

FP13 TN

So, you need, you, probably, want all data information to go into a central point in the practice... and then somebody to workflow it in the practice... otherwise if I go off on holiday for 3 weeks then it’s going to sit in my inbox for 3 weeks... and nothing’s going to happen.

FP7 TE

There was concern that telehealth integration could potentially disrupt existing plans of care. Consequently, interdisciplinary communication and the development of service agreements, as to who dealt with what information and how this was communicated to the entire team, were seen as essential to the implementation and safe delivery of the service.

But it seems that there could become this situation where we're getting results, the specialist nurses are already going in and there could be this scenario of who's dealing with what?

FP3 TN

Several physicians and nurses suggested that ensuring data (which did not require emergency action) that reached the appropriate case manager could be assisted by normalization within the current data flows to assimilate everyday dealings with existing data sources such as laboratory results or hospital letters. Existing practice flows included a central practice inbox, visibility of abnormal results in clinician’s workflow and individual records simultaneously, and the ability to feedback to other practice staff and care teams for any actions taken based on these results without adding excessively to the clinical workload. A model pathway based on these discussions is shown in Figure 1.

###### Data Coding

Another important design feature was enabling imported data to not only appear on the consultation page, but also simultaneously populate the clinical records result pages, including graphs. Presentation of data needed to be clearly delineated from other information, and imported data should not occupy much space on the consultation screen. For example, it was suggested that incoming telehealth messages should be visually distinct from other messages in the inbox and state exactly what data are enclosed. This would be achieved by ensuring data entries were appropriately coded and it was suggested that codes should allow differentiation of telehealth readings from readings taken in the practice or elsewhere (linked color coding or annotation).

I think it’ll be quite important to somehow integrate it so that if you wanted to see all the blood pressures they would all be there regardless of where they were taken, so that it’s not a completely separate system but that you would actually have everything at a glance. At the same time though, be it with a different color or a note or something, to say that they were from a different system or home readings.

FP12 TN

Color was also thought to help information processing. Highlighting normal parameters on the graph as shaded or colored was suggested; these should ideally be values set for specific patients and not generic.

Color just because, like I said previously, there’s so much to read, there’s so much to do… Certainly, the graph’s good because it’s instant and you can—it instantly tells you we need to look at that.

PN11 TN

##### Reimbursement

Clinicians identified financial incentive as a strong driver toward implementing an integrated system. Some thought that additional financial incentives were required to persuade family physicians to adopt the system, and for taking on additional workload. However, it was also recognized that in the United Kingdom, data recording to demonstrate the achievement of targets in the management of long-term conditions is already financially incentivized under the Quality and Outcomes Framework (QOF) [27]. The integrated system had the potential to facilitate this.

I mean that sounds a bit, blunt but yes, it [family practice] is a business and they [family physicians] are always looking for ways to make extra cash…

PN19 TN

If you market it from a QOF point, I think every GP practice would take it on. ….. Just always market it as that, it's going to help you get your QOF points.

PN18 TE

#### Safety

##### Professional-Patient Relationship

Positively, it was considered that integrated delivery of care may encourage self-management and mutual respect between clinicians and patients. The integrated system was unlikely to influence clinicians’ manner, as most have adapted to computers within their patient consultation. However, concern over moving toward a data-focused approach was raised including potentially missing clinical cues and interpreting data in a vacuum.

I suppose, general practice, I suppose, always focuses on people, patients, persons, real people, and of course recordings are part of a picture, but I think you have to be careful you don’t get caught up with what the machine is saying you feel, rather than actually how you do feel, and I think there is a wee danger, if you become too focused simply on measurements, actually that becomes your goal.

FP1 TN

##### Data Quality

Participants identified risks associated with patient self-monitoring, their competence in taking readings, the resultant quality of data, and the implications of integrating the data permanently into clinical records. Ways of handling these concerns included ensuring patients’ received good training that their equipment and techniques are reviewed.

All of a sudden you’ve got a reading which makes no clinical sense. If you could somehow remove that or put it there but not actually making it count with a reason for it… you can’t delete them, you just have to put a comment on it….

FP12 TN

##### Risks of Data Overload and Error

Increased workload was predicted in checking incoming results and also “actioning” anything abnormal, and work overload risked negative consequences on care.

I don’t know... if you’re inundated with too much information, and it’s all normal information then the ones that need acting on might, it’s easier to miss them.

FP17 TN

##### Confidentiality

There was acknowledgement among participants that confidentiality of data could be a risk associated with integration of telehealth data into the EMR; however, greater concern was expressed over the confidentiality of current paper-based results and the Web-based telehealth system.

Protective factors were the existence of health care professionals’ codes of conduct and obtaining informed consent from patients.

The other thing is the website that we use at the moment, I’m not sure how safe it is, from an information governance point of view, and I think that’s been a bit of the issues about populating it with more patient data.

Physical therapist (PT)4 TE

##### Liability

Medicolegal liability of integrating telehealth data was considered a risk by some participants. However, in contrast to our expectations, most participants felt that the integrated system was not different from any other results and would not place them at increased medicolegal risk; in fact it may even be protective.

I think that would be a massive concern, and I think particular of one of my partners would be absolutely catatonic looking at this, don't send me things unless you want me to take responsibility. And I think it's that thing of the collusion of anonymity if loads of people are getting results who exactly is dealing with it?

FP3 TN

But it’s like anything, any result that comes through, you know, from that point of view, you’ve, the minute it lands in your docman [laboratory result management system], or on your desk then that’s you, you’ve got to sort it out, haven’t you, so.

PN15 TE

Textbox 2 presents a summary of clinician recommendations arising from these in-depth interviews and Figure 1 shows a model of how data might be integrated with minimal disruption to current data management pathways.

**Summary of clinician recommendations.**

1. Any system must be simple and compatible with existing EMR system
2. Clear lines of responsibility must be agreed in terms of who must make the first response to abnormal results. This is likely to differ by the condition monitored
3. Lead carers would receive and deal with “action” data reports and the other care team members would only require much less regular “information-only” summaries
4. Data flows should be normalized to as closely resemble existing incoming data flows as possible (for proposed pathway, see Figure 1) and include a mechanism to feedback information to other care team members
5. Minimizing the amount of imported data is essential and screen filters may be useful
6. Graphical presentation and the use of color are helpful to summarize data and indicate data parameters; however, easy access to an attachment of the full dataset from a summarized chart is extremely useful
7. Flexibility of data alert parameters is beneficial only if they are easy to set
8. Coding of incoming telehealth data to identify which data are patient accrued (possibly color coded) is desirable.
9. Training should involve the instruction of several “on-site” experts who can assist other practice members and IT support, both in practice and from the software company, need to be easy to access
10. Gradual introduction of any new system, initially with small numbers of patients/conditions

## Discussion

### Principal Findings

Our study showed that participants were generally very positive about prototype software designed to improve integration of telehealth data with the EMR and were eager to explain what aspects of the system would increase its acceptability and facilitate its use. System design, in particular, was explored in detail, which enabled the design of a proposed data pathway modeled on clinicians’ preferences (Figure 1) and a list of recommendations to aid implementation of such software (Textbox 2). The key factors were ease of use; receiving as little incoming data as clinically necessary, the normalization of data flows, and ensuring clear lines of communication and responsibility for different clinicians involved in the care of the patient. Liability concerns while expressed were not a major issue, nor were concerns regarding the reliability of the patient accrued data.

### Strengths and Limitations

A strength of our study was that the project team came from a mixed background of clinical, research, and IT experience that provided rounded understanding and input into the creation of the coding framework. In addition, the research fellow had broad experience as a clinician, researcher, and public health specialist; thus perhaps enabling more frank discussion as the researcher appreciated the context in which the interviewees were working and the way in which this technology may interact with their working practices.

While we were successful in recruiting a range of clinicians from both telehealth naïve and experienced practices in a range of practice size and deprivation, it may be that those expressing an interest in this type of study were more interested in technology than the general population. As in previous studies the use of demonstration software helped stimulate discussion; however, the ability to interact with the software in a “live” situation would have been preferable. The EMIS EMR software was unfamiliar to some using alternative EMR software and this may have reduced their ability to see the full potential of the integrative software. Finally, the research was carried out in only one country which raises potential issues in respect of transferability.

### Comparison With Prior Work

Our results are in keeping with the literature—including the normalization process model (NPM), which has been established as a useful framework in considering introducing telehealth care for chronic conditions [28]. Normalization has been defined as “an ongoing cycle of activity aimed at making a new practice ‘fit in’ with the work of individuals and their context of practice” [29]. Our overarching theme “perceptions of workload” reflected the NPM dimension of “interactional workability” in terms of how the work would take place and whether the telehealth innovation would increase or decrease the ease and efficiency of their work. This theme also incorporated elements of “contextual integration” in terms of how the health care organizations may provide resources to reimburse the additional time and effort required by the telehealth innovation. Our other overarching theme of “safety” relates to the concepts of “relational integration” and “skill set workability” in terms of how the telehealth care system may alter the health care team relationships, division of labor, boundaries of practice, accountability, and confidence in the safety of the system [30].

Our findings also reflected the broader literature on conditions that influence clinicians’ decision to adopt or reject innovations in health care settings in that we identified the influence of “system antecedents” on the adoption of an integrated system [19,23,31]. Participants’ prior experience with telehealth care particularly acted as a driver toward an integrated system as they understood difficulties with the existing system, could visualize the potential of integration, and this overcame resistance to change [32]. Ease of use of the system was another important driver to adoption which is a common feature of several existing models of information technology acceptance [33] and is defined as “the degree to which a person believes that using a particular system would be free of effort” [34]. Additional drivers to adoption were identified as enhanced patient care, confidentiality, and financial benefit.

Likely barriers to adoption were participants’ unfamiliarity with IT, negative experience with implementation of preceding IT systems, and particularly the compatibility of the integrated system with their normal work practices and ethos. Compatibility of telehealth with health care delivery has previously been acknowledged as having an important role in determining telehealth adoption [35]. Furthermore, the need to ensure clear evidence-based care plans that inform decision making [16], and the importance of recognizing any additional workload which may arise as a result of non-face-to-face clinical encounters have been identified as challenges in multiple settings, including the emerging concept of the patient-centered medical home [17].

Other perceived challenges included workload, ensuring data quality and confidentiality, liability risks, and sustainability. Surprisingly, liability was not as strong a concern as has been suggested by previous studies [27,36,37] as many saw the additional data as no different from other sources of data with which they were used to dealing and taking responsibility for. The principal risk perceived by these clinicians was increased workload. If, however, the system was designed to accommodate their needs and usual practices, they could also see substantial benefits in terms of accessing and streamlining telehealth data, potentially reducing consultations and home visits and enhancing patient care.

Our recent systematic review of eHealth literature [1] identified that considerable changes to consultation dynamics and workflow processes can occur with the introduction of telehealth care. For an integrated system, the potential changes included more immediate patient demands and altered care pathways. Consequently, participants emphasized the importance of interdisciplinary communication and service agreements to delineate roles and responsibilities in the delivery of care.

### Conclusions

There is a growing evidence base informing deliberations on the use of telehealth to manage long-term conditions. A key success consideration is how the technology integrates into routine practice and for this to happen it must be seen as both easy to use and effective. The lack of integration of telehealth data with the EMR has been a source of frustration for the physicians and nurses attempting to use these systems in trial contexts [12,14]. Our study has demonstrated the potential acceptability and clinical utility of a telehealth integrated system among primary care clinicians, with specific caveats strongly expressed by the participants to ensure compatibility with existing care practices and normalization of data flows. Our work has provided clear pointers to the system design preferred by clinicians and should therefore contribute to future systems development as telehealth care moves from an experimental phase to a technology that is embedded into routine models of care delivery.

## Multimedia Appendix 1

Description and screenshots from EMIS.

## Multimedia Appendix 2

Topic guide.

## Multimedia Appendix 3

Characteristics of participants.

## Abbreviations

BP: blood pressure
CHF: congestive heart failure
COPD: chronic obstructive pulmonary disorder
EMR: electronic medical records
FP: family physician
IT: information technologies
NPM: normalization process model
PN: practice nurse
PT: physical therapist
QOF: quality and outcomes framework
RCTs: randomized controlled trials
TE: telehealth care experienced
TN: telehealth care naïve

## References

[1] McLean S, Protti D, Sheikh A (2011). Telehealthcare for long term conditions. BMJ.

[2] Polisena J, Tran K, Cimon K, Hutton B, McGill S, Palmer K, Scott RE (2010). Home telemonitoring for congestive heart failure: a systematic review and meta-analysis. J Telemed Telecare.

[3] Polisena J, Tran K, Cimon K, Hutton B, McGill S, Palmer K (2009). Home telehealth for diabetes management: a systematic review and meta-analysis. Diabetes Obes Metab.

[4] McKinstry B, Hanley J, Wild S, Pagliari C, Paterson M, Lewis S, Sheikh A, Krishan A, Stoddart A, Padfield P (2013). Telemonitoring based service redesign for the management of uncontrolled hypertension: multicentre randomised controlled trial. BMJ.

[5] McLean S, Nurmatov U, Liu JL, Pagliari C, Car J, Sheikh A (2012). Telehealthcare for chronic obstructive pulmonary disease: Cochrane Review and meta-analysis. Br J Gen Pract.

[6] McLean S, Chandler D, Nurmatov U, Liu J, Pagliari C, Car J, Sheikh A (2010). Telehealthcare for asthma. Cochrane Database Syst Rev.

[7] May C, Harrison R, Finch T, MacFarlane A, Mair F, Wallace P, Telemedicine Adoption Study Group (2003). Understanding the normalization of telemedicine services through qualitative evaluation. J Am Med Inform Assoc.

[8] Cresswell KM, Bates DW, Sheikh A (2013). Ten key considerations for the successful implementation and adoption of large-scale health information technology. J Am Med Inform Assoc.

[9] Broens TH, Huis in't Veld RM, Vollenbroek-Hutten MM, Hermens HJ, van Halteren AT, Nieuwenhuis LJ (2007). Determinants of successful telemedicine implementations: a literature study. J Telemed Telecare.

[10] Cresswell K, Morrison Z, Crowe S, Robertson A, Sheikh A (2011). Anything but engaged: user involvement in the context of a national electronic health record implementation. Inform Prim Care.

[11] The Telescot Programme.

[12] Hanley J, Ure J, Pagliari C, Sheikh A, McKinstry B (2013). Experiences of patients and professionals participating in the HITS home blood pressure telemonitoring trial: a qualitative study. BMJ Open.

[13] Fairbrother P, Ure J, Hanley J, McCloughan L, Denvir M, Sheikh A, McKinstry B, The Telescot programme team (2013). Telemonitoring for chronic heart failure: the views of patients and healthcare professionals---a qualitative study. J Clin Nurs.

[14] Ure J, Pinnock H, Hanley J, Kidd G, McCall Smith E, Tarling A, Pagliari C, Sheikh A, MacNee W, McKinstry B (2012). Piloting tele-monitoring in COPD: a mixed methods exploration of issues in design and implementation. Prim Care Respir J.

[15] Alexandru CA, McKinstry B (2012). Usability evaluation of clinician web back-ends to telemonitoring systems: two case-studies in Scotland. Stud Inform Control.

[16] Berenson RA, Hammons T, Gans DN, Zuckerman S, Merrell K, Underwood WS, Williams AF (2008). A house is not a home: keeping patients at the center of practice redesign. Health Aff (Millwood).

[17] Kellerman R, Kirk L (2007). Principles of the patient-centered medical home. Am Fam Physician.

[18] Ackerman MJ, Filart R, Burgess LP, Lee I, Poropatich RK (2010). Developing next-generation telehealth tools and technologies: patients, systems, and data perspectives. Telemed J E Health.

[19] Rogers EM (2003). Diffusion of Innovations. 5th edition.

[20] Sheikh A, Cornford T, Barber N, Avery A, Takian A, Lichtner V, Petrakaki D, Crowe S, Marsden K, Robertson A, Morrison Z, Klecun E, Prescott R, Quinn C, Jani Y, Ficociello M, Voutsina K, Paton J, Fernando B, Jacklin A, Cresswell K (2011). Implementation and adoption of nationwide electronic health records in secondary care in England: final qualitative results from prospective national evaluation in "early adopter" hospitals. BMJ.

[21] Philips Motiva.

[22] EMIS.

[23] Greenhalgh T, Robert G, Macfarlane F, Bate P, Kyriakidou O (2004). Diffusion of innovations in service organizations: systematic review and recommendations. Milbank Q.

[24] Pope C, Ziebland S, Mays N (2000). Qualitative research in health care. Analysing qualitative data. BMJ.

[25] Eisenhardt K (1989). Building theories from case study research. Acad Manage Rev.

[26] Fairbrother P, Pinnock H, Hanley J, McCloughan L, Sheikh A, Pagliari C, McKinstry B (2013). Exploring telemonitoring and self-management by patients with chronic obstructive pulmonary disease: a qualitative study embedded in a randomized controlled trial. Patient Educ Couns.

[27] The National Institute for Health and Care Excellence. About the Quality and Outcomes Framework (QOF).

[28] Mair FS, Hiscock J, Beaton SC (2008). Understanding factors that inhibit or promote the utilization of telecare in chronic lung disease. Chronic Illness.

[29] Finch TL, Mair FS, O'Donnell C, Murray E, May CR (2012). From theory to 'measurement' in complex interventions: methodological lessons from the development of an e-health normalisation instrument. BMC Med Res Methodol.

[30] May C, Finch T, Mair F, Ballini L, Dowrick C, Eccles M, Gask L, MacFarlane A, Murray E, Rapley T, Rogers A, Treweek S, Wallace P, Anderson G, Burns J, Heaven B (2007). Understanding the implementation of complex interventions in health care: the normalization process model. BMC Health Serv Res.

[31] Cresswell K, Sheikh A (2013). Organizational issues in the implementation and adoption of health information technology innovations: an interpretative review. Int J Med Inform.

[32] Clark M, Goodwin N Sustaining innovation in telehealth and telecare.

[33] Venkatesh V, Morris MG, Davis GB, Davis FD (2003). User acceptance of information technology: toward a unified view. MIS Quarter.

[34] Davis FD (1989). Perceived usefulness, perceived ease of use, and user acceptance of information technology. MIS Quarter.

[35] Vuononvirta T, Timonen M, Keinänen-Kiukaanniemi S, Timonen O, Ylitalo K, Kanste O, Taanila A (2011). The compatibility of telehealth with health-care delivery. J Telemed Telecare.

[36] Anderson JG (2007). Social, ethical and legal barriers to e-health. Int J Med Inform.

[37] Catwell L, Sheikh A (2009). Evaluating eHealth interventions: the need for continuous systemic evaluation. PLoS Med.

