# Supplementary material for: Integrating Telehealth Care-Generated Data With the Family Practice Electronic Medical Record: Qualitative Exploration of the Views of Primary Care Staff
Source: Interact J Med Res. 2013 Nov 26;2(2):e29. doi: 10.2196/ijmr.2820 (PMC3869047; doi:10.2196/ijmr.2820)

Appendix 1 Description and screenshots from EMIS/Philips software system

This pilot software was developed the UK Department of Health as a proof of concept project focussing on integration of telehealth accrued data with Primary care electronic medical records. In this case, telehealth data from the Philips Motiva telehealth technology was integrated into general practice electronic health records held in EMIS web. The standards basis of this demonstrator solution means that the specifications and supporting rules are openly shareable across the clinical ICT industry and are not the exclusive intellectual property of Philips or EMIS. The specifications are compliant with the standards proposed by the Continua Health Alliance, Integrating the Healthcare Enterprise (IHE®) and implemented by NHS England's National Programme for IT and work is being undertaken by all these organisations to increase the adoption of these standards across global healthcare.

Incoming telehealth reports. Report opened to view

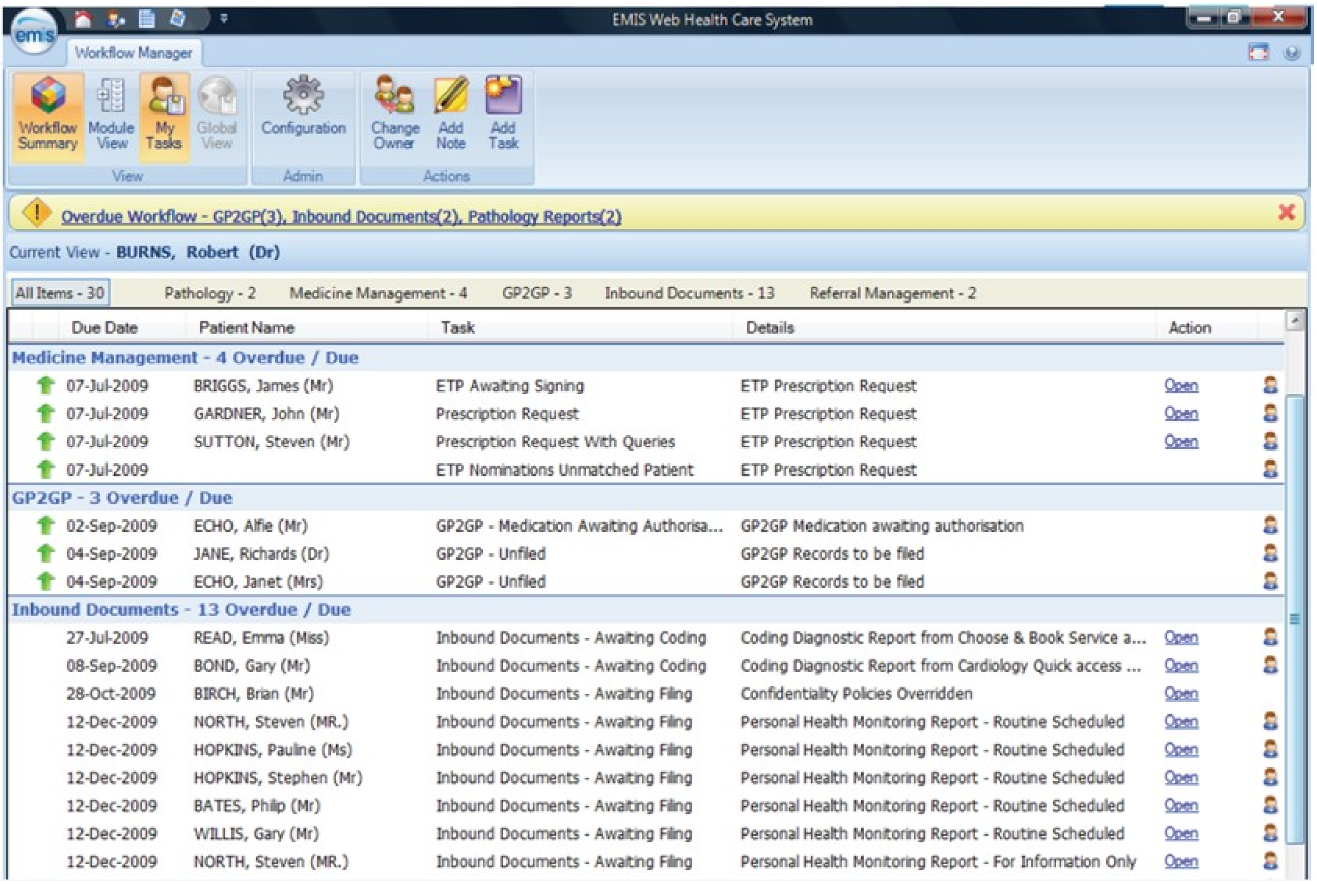

Report including graphical data

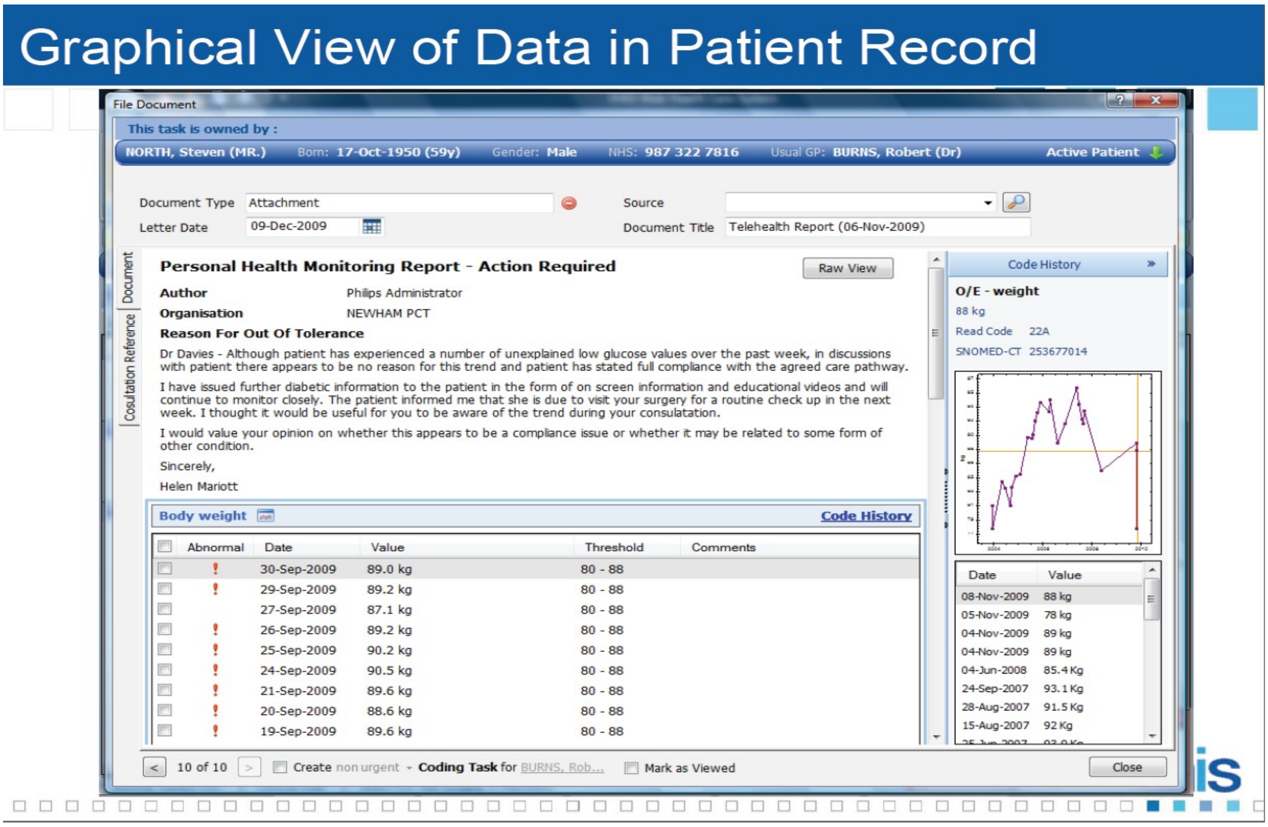

Supplement: Supplementary file 1 [file ijmr_v2i2e29_app1.pdf]
