# Supplementary material for: Integrating Telehealth Care-Generated Data With the Family Practice Electronic Medical Record: Qualitative Exploration of the Views of Primary Care Staff
Source: Interact J Med Res. 2013 Nov 26;2(2):e29. doi: 10.2196/ijmr.2820 (PMC3869047; doi:10.2196/ijmr.2820)
Supplement: Supplementary file 2 [file ijmr_v2i2e29_app2.pdf]

Appendix 2 Topic Guide

Main topic	Possible issues for exploration
Demonstrate integrated system	• answer and discuss any questions which arise from the demonstration
Familiarity	• what systems are they currently working with for patient records and for receiving pathology/radiology results<br>• have they experience in working with patient acquired data in this practice or elsewhere
Advantage	• does the software offer a relative advantage (over the current standalone system)<br>• what benefits are readily apparent<br>• does it appear simple to use<br>• do they think the software offers the opportunity to experiment with how it's used<br>• what other advantages are apparent
Disadvantages	• what are the apparent disadvantages of the system<br>• what are the risks perceived in implementing this software<br>• what are the medico-legal issues in implementing this software<br>• what other concerns would they have about this system
System design	• would it be preferable to have the patient recorded data added to their medical records (as in this system) or to have patient details on the Telehealth website- explore medico-legal aspects of these options<br>• how frequently would they like the data to be uploaded<br>• what kind of data and what detail of data would they like e.g. every value, an average, or a summary sheet (what would be required in a summary)<br>• is numerical or graphical data preferable<br>• would they want alerted of abnormal/especially high risk values<br>• what data quality aspects are important to them<br>• Do they have concerns of speed of access of information<br>• what other aspects of the design would they prefer or change
Compatibility	• how relevant is the system to the practice staff<br>• how compatible is the software with the values, norms and perceived needs of the practice staff<br>• how likely would they be to adopt using this system<br>• how malleable is the system in terms of allowing users to adapt and refine to their own needs and circumstances<br>• how might this system affect their delivery of care and their consultation/communication<br>• would it be useful to have this information more at hand when consulting with patients – are there any drawbacks

	to having it more accessible (patient expectations)<br>• how might this system affect their work schedule/ work load both within and out with consultation
Service agreements	• what items would be important for them to include in service agreements e.g. data limits, alerts, processes for follow up, tracking of data
Training and support	• what training would be required to adopt such a system<br>• what range of support would be required to help with the adoption of the software
Confidentiality	• what concerns might there be of the confidentiality of data
Recommendations	• what recommendations do they have for the system
