# Supplementary material for: Integrating Telehealth Care-Generated Data With the Family Practice Electronic Medical Record: Qualitative Exploration of the Views of Primary Care Staff
Source: Interact J Med Res. 2013 Nov 26;2(2):e29. doi: 10.2196/ijmr.2820 (PMC3869047; doi:10.2196/ijmr.2820)
Supplement: Supplementary file 3 [file ijmr_v2i2e29_app3.pdf]

Appendix 3 Participant characteristics

Interview number	*Profession*	*Telehealth experience*	*Gender*
1	*FP*	*No (had in practice but not involved)*	*F*
2	*FP*	*No*	*F*
3	*FP*	*No (had in practice but not involved)*	*F*
4	*PT*	*Yes*	*F*
5	*FP*	*No*	*M*
6	*PT*	*Yes*	*F*
7	*FP*	*Yes*	*M*
8	*PN*	*Yes*	*F*
9	*PN*	*Yes*	*F*
10	*FP*	*Yes*	*M*
11	*PN*	*No*	*F*
12	*FP*	*No*	*F*
13	*FP*	*No*	*F*
14	*PN*	*Yes*	*F*
15	*PN*	*Yes*	*F*
16	*FP*	*Yes*	*F*
17	*FP*	*No*	*F*
18	*PN*	*Yes*	*F*
19	*PN*	*No*	*F*
20	*PN*	*No*	*F*

FP family clinician,PN practice nurse, PT Physical Therapist
